# Application of the diastereoselective photodeconjugation of α,β-unsaturated esters to the synthesis of gymnastatin H

**DOI:** 10.3762/bjoc.7.21

**Published:** 2011-02-02

**Authors:** Ludovic Raffier, Olivier Piva

**Affiliations:** 1 Université Lyon 1, UMR 5246 CNRS, Institut de Chimie et de Biochimie Moléculaire et Supramoléculaire, 69622 Villeurbanne, France

**Keywords:** amide, diastereoselective protonation, dienol, isomerisation, Wittig reaction

## Abstract

The asymmetric synthesis of gymnastatin H has been achieved by using the photoisomerisation of a conjugated ester to its β,γ-unsaturated isomer through the protonation of a in situ generated dienol as key step. Thanks to diacetone D-glucose used as a chiral alkoxy group, the protonation occurred well onto one of the two diastereotopic faces with very high yields and selectivities. Moreover, by this way the configuration of the C-6 centre of the target molecule was controlled.

## Introduction

**Scheme 1:**
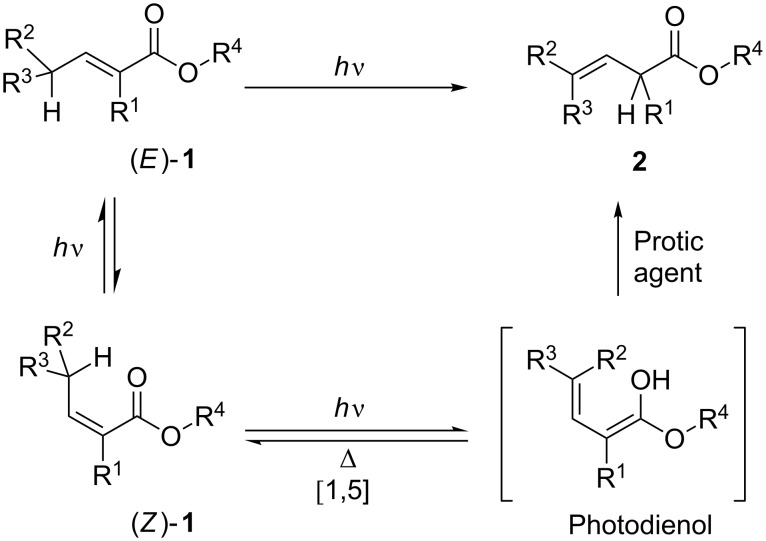
Principle of the photodeconjugation process.

The photodeconjugation of α,β-unsaturated esters **1** – which bear at least one hydrogen atom on γ-position – allows a straightforward access to β,γ-unsaturated isomers **2** [1]. This reaction was first reported by Jorgenson [2–3] and has been extensively studied by different groups in a mechanistical point of view. For example, Weedon et al. were able to trap an intermediate species identified as a photodienol (its formation resulting from a [1,5]-sigmatropic rearrangement). It was shown that the efficiency of the isomerisation process is highly dependent on the nature of the solvent and on the presence of various additives (e.g., amines) which could catalyse the reketonisation of the transient dienol [4] (Scheme 1).

Despite the potential interest in β,γ-unsaturated acid derivatives, until recently only a few applications of this photochemical transformation in total synthesis appeared in the literature.

**Scheme 2:**
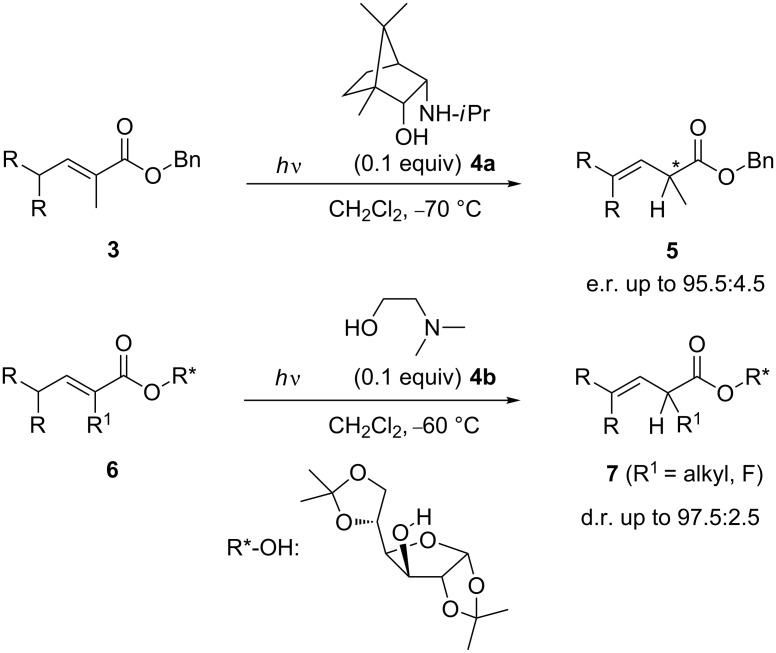
Enantio- and diastereoselective photodeconjugation reactions.

**Figure 1:**
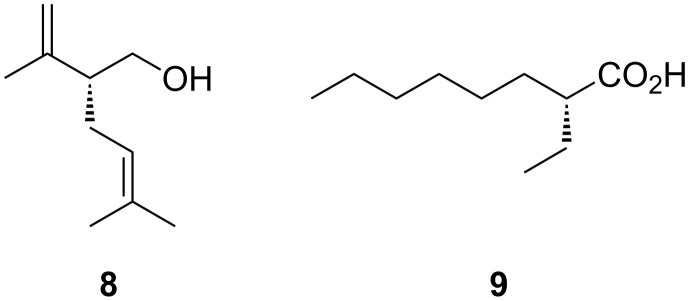
Natural products prepared by photodeconjugation.

However, the scope of the photochemical isomerisation has been greatly enhanced thanks to the development of diastereo- and enantioselective versions. Starting from α-substituted esters **3** in the presence of a catalytic amount of an enantiomerically pure bicyclic amino alcohol **4** – derived from camphor –, the protonation of the prochiral photodienol can be achieved with an ee up to 91% [5]. This value is one of the highest values observed for an enantioselective protonation transformation reaction. However, as the selectivities were highly dependent on the substrate, an alternative diastereoselective version has been developed. By using cheap and commercially available diacetone D-glucose (DAG-OH) as chiral alkoxy group and dimethylamino alcohol as additive, a selective protonation of one of the two diastereotopic faces of the transient dienol was achieved which lead to esters **7** with a d.r. better than 97.5:2.5 [6] (Scheme 2). This transformation allowed the formation of a new allylic stereogenic centre and found already a direct application to the asymmetric synthesis of different natural products including *(R)*-lavandulol (**8**), *(R)*-arundic acid (**9**) and 2-fluoroacids or lactones [7–9] (Figure 1).

**Figure 2:**
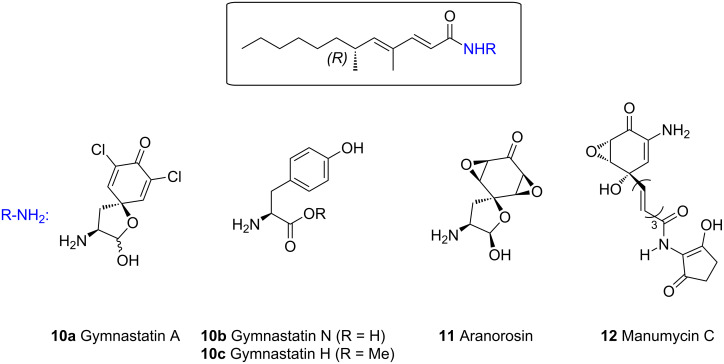
Natural amides possessing the same (6*R*)-fatty acid side chain.

Filamentous fungi are the source of a wide range of secondary metabolites which possess very promising biological activities. Among them, gymnastatins **10** constitute a family of compounds isolated from *Gymnascella dankaliensis* which grows in symbiosis with the marine sponge *Halichondria japonica* [10] (Figure 2).

Gymnastatins **10** possess a common unsaturated fatty acid residue connected to a tyrosine subunit. These compounds have been reported to exhibit antibacterial activity and cytotoxities against cultured P388 cancer cells. Interestingly, the same acid chain with an *R*-configuration has been identified in other structures like dankastatins [11] isolated from the same source, aranorosin (**11**) isolated from *Pseudoarachniotus roseus* [12] and manumycin C (**12**) isolated from *Streptomyces parvulus* [13]. Different groups have investigated the synthesis of gymnastatins **10a**–**c** [14–16], compounds **11** [17] and **12** [18]. In most cases, the lateral acid chain was prepared starting from *(R)*-2-methyloctanal by iterative Wittig reactions to build the dienoate chain. The configuration at the C-2 carbon atom of this precursor was controlled by using a diastereoselective alkylation of an acyl oxazolidinone. In some cases, a Claisen condensation took place and afforded a β-ketoamide in noticeable amounts diminishing the overall yield of the sequence [15]. In this context, we have considered an alternative synthetic route to the fatty acid common to all gymnastatins according to a photoisomerisation–diastereoselective protonation sequence involving catalytic amounts of an achiral organocatalyst (e.g., amino alcohol **4b**). Our goal was to describe the first de novo total synthesis of gymnastatin H (**10c**).

## Results and Discussion

**Scheme 3:**
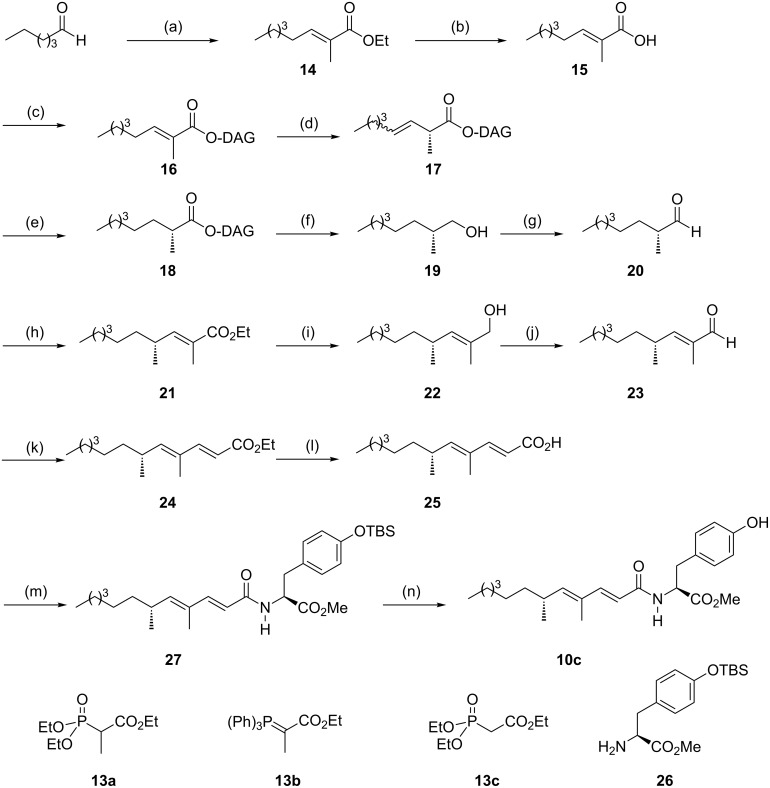
Reagents and conditions: (a) NaH, **13a**, THF, 25 °C, 83%. (b) KOH, EtOH/H_2_O (95/5), Δ, 97% (*E*/*Z*: 90/10). (c) DAG-OH, DCC, DMAP, CH_2_Cl_2_, 0 °C then rt, 76%. (d) *h*ν (254 nm), **4b** (0.3 equiv), CH_2_Cl_2_, −60 °C, 90%. (e) H_2_ (1 atm), PtO_2_ (cat.), Et_2_O, 99%. (f) LiAlH_4_, Et_2_O, 0 °C, 83%. (g) DMP, CH_2_Cl_2_, 0 °C, 70%. (h) **13b**, PhMe, 80 °C, 80% (*E*-only). (i) Dibal-H (2 equiv), THF, 0 °C, 99%. (j) DMP, CH_2_Cl_2_, 0 °C, 75%. (k) **13c**, NaH, THF, rt, 60%. (l) LiOH, MeOH, THF, H_2_O, 70%. (m) **26**, DCC, HOBt, CH_2_Cl_2_, 57%. (n) TBAF, THF, 0 °C, 96%.

Ethyl ester **14,** readily prepared from hexanal by a Wittig–Horner reaction, was saponified and esterified under DCC activation with commercially available diacetone D-glucose (Scheme 3). Irradiation of **16** at 254 nm in methylene chloride at −60 °C delivered the β,γ-unsaturated ester **17** in 90% yield as an inseparable mixture of *E*- and *Z*-isomers. Hydrogenation of the double bond lead to the saturated ester **18** for which a 95:5 diastereomeric ratio was measured by ^1^H NMR spectroscopy. Next, a two-step sequence delivered 2-methyloctanal (**20**) in 58% overall yield. The configuration of the newly created centre was first assigned as *R* by applying a model we disclosed earlier and was confirmed by comparison with optical rotation values published in the literature [19]. Aldehyde **20** was submitted to a Wittig condensation with phosphorane **13b** at reflux of toluene to deliver ester **21** only as the *E*-isomer. It should be pointed out that the Wadsworth–Emmons variant using 2-phosphonatoester **13a** led mainly to the *Z*-isomer, a phenomenon which was already observed with α-substituted aldehydes [20]. The reduction of the ethyl ester into the corresponding allylic alcohol **22** followed by the oxidation with Dess–Martin periodinane (DMP) [21] afforded aldehyde **23** which was converted into the known ethyl ester *(E,E)*-**24** by a subsequent Wittig–Horner reaction. The comparison of the optical rotation with literature data confirmed the (*R*) configuration at the C-6 carbon. By saponification under mild conditions, **24** was converted into carboxylic acid **25** which was implicated into a free-epimerising amidation procedure with HOBt [22] and the readily available *O*-protected tyrosine derivative **26**. Finally, the TBS group of the amino ester moiety was removed under standard conditions to deliver compound **10c**. The measured spectroscopic data were identical to those reported for gymnastatin H [10]. Interestingly, the optical rotation of the synthetic product showed a higher value ([α]_D_^25^ = +104 (0.3, CHCl_3_)) than those measured for the isolated natural product ([α]_D_^25^ = +42.3 (0.76, CHCl_3_)). Similar discordances have been already observed in the case of gymnastatin N and were shown to be a consequence of a partial epimerisation at the C-2’ carbon of the natural compound's amino ester subunit [15].

## Conclusion

In conclusion, we have achieved the total synthesis of (6*R*)-gymnastatin H in 14 steps and 4.3% overall yield by using a highly diastereoselective photodeconjugation of a diacetone D-glucose ester as key step (de >95%). Now, work is underway to prepare parent structures that either possess an opposite configuration on the stereogenic centre or a modified geometry of the two double bonds. Furthermore, the biological activities of these novel structures are going to be studied.

## Supporting Information

File 1 Full experimental and spectral data for compounds **10c**, **14**–**27**.
