# Supplementary material for: Application of the diastereoselective photodeconjugation of α,β-unsaturated esters to the synthesis of gymnastatin H
Source: Beilstein J Org Chem. 2011 Feb 2;7:151–5. doi: 10.3762/bjoc.7.21 (PMC3064449; doi:10.3762/bjoc.7.21)
Supplement: File 1 — Full experimental and spectral data for compounds 10c, 14–27. [file Beilstein_J_Org_Chem-07-151-s001.pdf]

Supporting Information

for

Application of the diastereoselective photodeconjugation of α,β-unsaturated esters to the synthesis of gymnastatin H

Ludovic Raffier and Olivier Piva*

Address: Université Lyon 1, UMR 5246 CNRS, Institut de Chimie et de Biochimie Moléculaire et Supramoléculaire, 69622 Villeurbanne, France.

E-mail: Olivier Piva* - piva@univ-lyon1.fr

*Corresponding author

Full experimental and spectral data for compounds 10c, 14–27

Ethyl 2-methyl-2-octenoate: 14 [1]

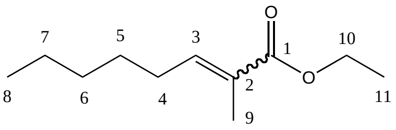

Triethyl 2-phosphonopropionate **13a** (11.98 mmol; 2.85 g; 1.2 equiv) was added dropwise to a suspension of NaH 60% weight in mineral oil (11.98 mmol; 479 mg; 1.2 equiv) in dry THF (100 mL) at 0 °C under an argon atmosphere. After 1 h stirring, hexanal (9.98 mmol; 1 g) was added. The reaction was stirred to rt for 2 h before being quenched with a saturated aqueous solution of NH_4Cl. The solvent was removed by half in vacuo and the resulting mixture was extracted with DCM (3 × 30 mL). The combined organic extracts were dried over $MgSO_4$, filtered and concentrated in vacuo. Flash chromatography (98:2 petroleum ether:EtOAc) afforded the known product **14** as a colorless oil in 83% yield (8.28 mmol; 1.527 g; *E*/*Z* mixture 5/1).

^{1}H NMR ($CDCl_3$, 300 MHz)**:** 0.85–0.98 (m, 3H, H_8); 1.24–1.40 (m, 7H, $H_{6,7,11}$); 1.40–1.54 (m, 2H, H_5); 1.85 (d, J=1.5 Hz, 2.5H, H_{9E}); 1.91 (d, J = 1.3 Hz, 0.5H, H_{9Z}); 2.16 (q, J = 6.8 Hz, 1.7H, H_{4E}); 2.43 (q, J = 7.2 Hz, 0.3H, H_{4Z}); 4.18 (q, J = 7.0 Hz, 1.7H, H_{10E}); 4.19 (q, J = 7.1 Hz, 0.3H, H_{10Z}); 5.9 (tq, ^3J = 5.9 Hz, ^4J = 1.5 Hz, 0.17H, H_{3Z}) ; 6.78 (tq, ^3J = 7.5 Hz, ^4J = 1.3 Hz, 0.83H, H_{3E}). **^{13}C NMR (**$CDCl_3$, 75 MHz)**:** 12.3 (q, C_9); 14.0–14.3 (q, 2C, $C_{8,11}$); 22.6–28.3 - 28.7–31.6 (t, 4C, $C_{4\text{-}7}$); 60.3 (t, C_{10}); 127.7 (s, C_2); 142.4 (d, C_3); 168.3 (s, C_1).

2-Methyl-2-octenoic acid: 15

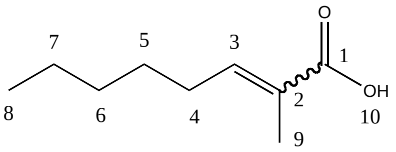

To a solution of **14** (7.5 mmol; 1.39 g) in EtOH/H_2O (50/10 mL) was added KOH (11.3 mmol; 633 mg; 1.5 equiv). The reaction was heated at reflux for 3 h. After being cooled down, the solution was concentrated by half in vacuo. The resulting mixture was diluted in ether and washed with a saturated aqueous solution of Na_2CO_3 (3 × 15 mL). The combined aqueous layers were acidified with 1N HCl until pH = 1 and then extracted with EtOAc (4 × 15 mL). The organic extracts were

dried over $MgSO_4$, filtered and concentrated in vacuo affording the desired product **15** as a pale yellow oil in 93% yield (6.97 mmol; 1.087 g; *E*/*Z* mixture 9/1).

^{1}H NMR ($CDCl_3$, 300 MHz)**:** 0.80–0.99 (m, 3H, H_8); 1.20–1.42 (m, 4H, $H_{6,7}$); 1.46 (m, 2H, H_5); 1.83 (s, 2.6H, H_{9E}); 1.91 (s, 0.4H, H_{9Z}); 2.19 (q, J = 7.3 Hz, 1.8H, H_{4E}); 2.51 (q, J = 7.1 Hz, 0.2H, H_{4Z}); 6.09 (tq, ^3J = 7.4 Hz, ^4J = 1.3 Hz, 0.1H, H_{3Z}); 6.92 (tq, ^3J = 7.4 Hz, ^4J = 1.1 Hz, 0.9H, H_{3E}).

(1,2,4,5-Di-*O*-isopropyliden-α-D-fructopyranose-3-*O*-yl) 2-methyl-2-octenoate : 16

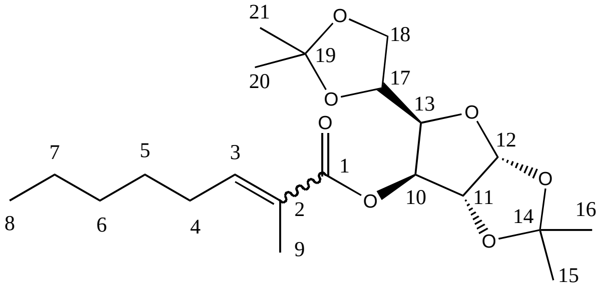

To a solution of **15** (6.96 mmol; 1.087 g), DMAP (2.09 mmol, 255 mg, 0.3 equiv) and diacetone D-glucose (7.66 mmol; 1.994 g; 1.1 equiv) at 0 °C in DCM (60 mL) was added DCC (7.66 mmol; 1.580 g; 1.1 equiv). The reaction was allowed to warm to rt overnight under stirring. The mixture was washed with water (2 × 20 mL) and the resulting aqueous layer was extracted with ether (3 × 15 mL). The combined organic extracts were dried over $MgSO_4$, filtered and concentrated in vacuo. Flash chromatography (90:10 petroleum ether:EtOAc) afforded the desired product **16** as a colorless oil in 77% yield (5.35 mmol; 2.123 g; *E*/*Z* mixture 5.7/1).

^{1}H NMR ($CDCl_3$, 300 MHz)**:** 0.77–0.91(m, 3H, H_8); 1.17–1.33 (m, 12H, $H_{5\text{-}7,15\text{-}16}$); 1.36 (s, 3H, H_{20}); 1.48 (s, 3H, H_{21}); 1.78 (s, 2.5H, H_{9E}); 1.99 (s, 0.5H, H_{9Z}); 2.13 (q, J = 7.3 Hz, 2H, H_4); 3.92–4.10 (m, 2H, H_{18}); 4.15–4.27 (m, 2H, $H_{13,17}$); 4.48 (d, J = 3.8 Hz, 1H, H_{11}); 5.23 (m, 1H, H_{10}); 5.84 (d, J = 3.8Hz, 1H, H_{12}); 6.73 (t, J = 7.5 Hz, 1H, H_3). **^{13}C NMR** ($CDCl_3$, 75 MHz)**:** 12.4–14.0 (q, 2C, $C_{8,9}$); 22.5–28.2–28.8–31.6 (t, 4C, $C_{4\text{-}7}$); 25.3–26.3–26.8 (q, 4C, $C_{15\text{-}16,\ 20\text{-}21}$); 67.3 (t, C_{18}); 72.7–76.3 - 80.1–83.4–105.2 (d, 5C, $C_{10\text{-}13,17}$); 109.3–112.2 (s, 2C, $C_{14,19}$); 127.1 (s, C_2); 144.2 (d, C_3); 166.8 (s, C_1).

(*2R*)-(1,2,4,5-Di-*O*-isopropyliden-α-D-fructopyranose-3-*O*-yl) 2-methyl-3-octenoate : 17

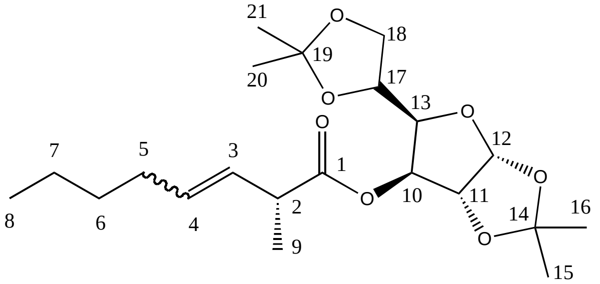

To a solution of **16** (0.9 mmol; 360 mg) in DCM (90 mL) was added *N*,*N*-dimethylethanolamine (0.9 mmol; 80.2 mg; 1 equiv). The resulting solution was degassed by bubbling argon and equally distributed in 6 quartz tubes which were irradiated (254 nm) at −60 °C for 6 h. The combined solutions were then passed without concentration through a silica pad then was washed with a 80:20 petroleum ether:EtOAc solution, affording after concentration in vacuo, the desired product **17** as a colorless oil in 91% yield (0.82 mmol; 326 mg; *E*/*Z* mixture 3/1) with a d.e. > 95% determined by ^{1}H NMR.

^{1}H NMR ($CDCl_3$, 300 MHz)**:** 0.84–0.94 (m, 3H, H_8); 1.24 (d, J = 7.0 Hz, 0.7H, H_{9Z}); 1.25 (d, J = 7.0 Hz, 2.3H, H_{9E}); 1.27–1.38 (m, 10H, $H_{6\text{-}7,15\text{-}16}$); 1.40 (s, 3H, H_{20}); 1.52 (s, 3H, H_{21}); 2.01 (q, J = 6.7 Hz, 1.5H, H_{5E}); 2.05 (q, J = 7.0 Hz, 0.5 Hz, H_{5Z}); 3.12 (q_t, J = 7.1 Hz, 0.7H, H_{2E}); 3.33–3.4 (m, 0.3H, H_{2Z}); 3.94–4.12 (m, 2H, H_{18}); 4.16–4.24 (m, 2H, $H_{13,17}$); 4.45 (d, J = 3.6 Hz, 1H, H_{11}); 5.25–5.26 (m, 1H, H_{10}); 5.32–5.63 (m, 2H, $H_{3\text{-}4}$); 5.87 (d, J = 3.8 Hz, 1H, H_{12}). **^{13}C NMR** ($CDCl_3$, 75 MHz) **:** 13.8–17.1 (q, 2C, $C_{8,9E}$); 17.7 (q, C_{9Z}); 22.1–31.2–32.0 (t, 3C, $C_{5E\text{-}7}$); 31.5 (t, C_{5Z}); 67.2 (t, C_{18}); 72.2–75.8 –80.0–83.3–105.0 (d, 5C, $C_{10\text{-}13,17}$); 109.1–112.1 (s, 2C, $C_{14,19}$); 128.1–132.7 (d, 2C, $C_{3E\text{-}4E}$); 127.9–132.2 (d, 2C, $C_{3Z\text{-}4Z}$); 173.2 (s, C_1). **$[\alpha]^{22}_D$** = -83.0 (c 1.9; $CHCl_3$). **HRMS:** Calculated for M + Na^+: 421.2202; Found M + Na^+: 421.2201.

(2*R*)-(1,2,4,5-Di-*O*-isopropyliden-α-D-fructopyranose-3-*O*-yl) 2-methyloctanoate: 18

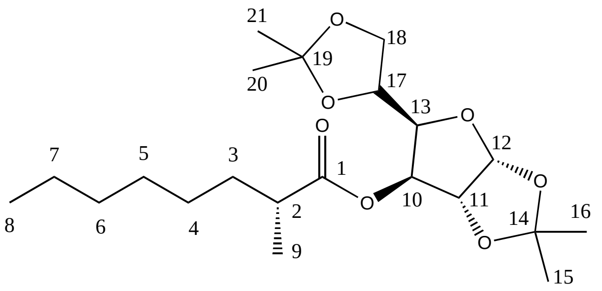

To a solution of **17** (2.4 mmol; 964 mg) in dry ether (20 mL) was added PtO_2 (0.12 mmol; 46 mg; 0.05 equiv). The reaction mixture was allowed to stir at room temperature for 6 h under a H_2 atmosphere. The catalyst was then removed by filtration and the resulting solution was concentrated in vacuo to afford the desired product **18** as a colourless oil in 99% yield (2.38 mmol; 952 mg).

^{1}H NMR ($CDCl_3$, 300 MHz)**:** 0.82–0.94 (m, 3H, H_8); 1.16 (d, *J* = 6.8 Hz, 3H, H_9); 1.23–1.35 (m, 14H, $H_{4\text{-}7,15\text{-}16}$); 1.40 (s, 3H, H_{20}); 1.52 (s, 3H, H_{21}); 1.56–1.75 (m, 2H, H_3); 2.46 (sext, *J* = 6.9 Hz, 1H, H_2); 3.96–4.15 (m, 2H, H_{18}); 4.17–4.27 (m, 2H, $H_{13,17}$); 4.44 (d, *J* = 3.8 Hz, 1H, H_{11}); 5.26 (d, *J* = 2.1 Hz, 1H, H_{10}); 5.87 (d, *J* = 3.6 Hz, 1H, H_{12}). **$[\alpha]^{22}_D$** = −26.0 (c 1.6; $CHCl_3$). **HRMS:** Calculated for M + Na^+: 423.2359; Found: M + Na^+: 423.2358.

(2*R*)-2-Methyloctanol: 19 [2]

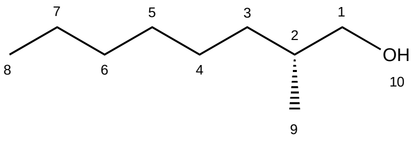

To a solution of **18** (2.4 mmol; 952 g) in dry ether (15 mL) at 0 °C, was slowly added $LiAlH_4$ (3.6 mmol; 135 mg; 1.5 equiv). The reaction was allowed to stir to rt for 1 h. The reaction was cooled down to 0 °C and quenched by adding dropwise and respectively water (140 µL), 1N NaOH (140 µL), and water again (280 µL). The formed precipitate was removed by filtration and the filtrate was concentrated in vacuo. Flash chromatography (90:10 petroleum ether:EtOAc) afforded the desired and known product **19** as a colorless oil in 83% yield (2.0 mmol; 288 mg) [2].

^{1}H NMR ($CDCl_3$, 300 MHz)**:** 0.82 (t, *J* = 7.0 Hz; 3H, H_8); 0.85 (d, *J* = 6.8 Hz, 3H, H_9); 1.08–1.13 (m, 1H, H_{3a}); 1.28–1.35 (m, 9H, $H_{4\text{-}7,3b}$); 1.61 (m, 1H, H_2); 3.46 (ddd, ^2J=28.4 Hz, $^3J_1$ = 10.3 Hz, $^3J_2$ = 5.6 Hz, 2H, H_1). **^{13}C NMR** ($CDCl_3$, 75 MHz)**:** 14.2 (q, C_8); 16.7 (q, C_9); 22.8–27.1–29.7–32.0–33.3 (t, 5C, $C_{3\text{-}7}$); 35.9 (d, C_2); 68.6 (t, C_1). **$[\alpha]^{22}_D$** = +5.0 (c 1.2; $CHCl_3$). Literature: **$[\alpha]^{22}_D$** = +10.3 (c 1.0; CH_2Cl_2) [1].

(2*R*)-2-Methyloctanal: 20 [3]

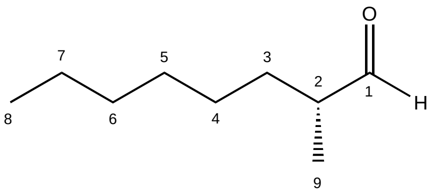

To a solution of **19** (1.88 mmol; 272 mg) in dry DCM (15 mL) at 0 °C was added the Dess-Martin periodinane 15% weight in DCM (2.26 mmol; 4.85 mL; 1.2 equiv). The reaction was allowed to stir to rt for 2.5 h and was then quenched by adding a 1:1 mixture of saturated aqueous NH_4Cl:$Na_2S_2O_3$. The aqueous layer was extracted with DCM (3 × 10 mL) and the combined organic extracts were dried over $MgSO_4$, filtered and concentrated in vacuo. Flash chromatography (95:5 petroleum ether:EtOAc) afforded the desired and known compound **20** as a colorless oil in 70% yield (1.32 mmol; 190 mg) [3].

^{1}H NMR ($CDCl_3$, 300 MHz)**:** 0.83–0.94 (m, 3H, H_8); 1.07 (d, *J* = 7.0 Hz, 3H, H_9); 1.27 (m, 9H, $H_{2\text{-}7,3a}$); 1.68 (m, 1H, H_{3b}); 2.32 (s_xd, J_1 = 6.8 Hz, J_2 = 2.1 Hz, 1H, H_2); 9.60 (d, *J* = 2.1 Hz, 1H, H_1). **$[\alpha]^{22}_D$** = −17.2 (c 1.9; $CHCl_3$).Literature [3]: **$[\alpha]^{22}_D$** = −19.7 (c 5.8; $CHCl_3$).

(*4R*)-Ethyl 2,4-dimethyl-2-decenoate: 21 [2]

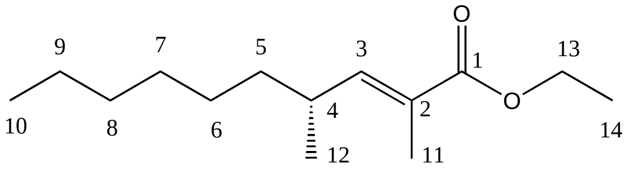

To a solution of **20** (0.86 mmol; 123 mg) in toluene (5 mL) was added the (carbethoxyethylidene) triphenylphosphorane (2.6 mmol; 1 g; 3 equiv). The reaction was heated at reflux for 16 h. After being cooled down, the solution was concentrated in vacuo. Flash chromatography (99:1 petroleum ether:EtOAc) afforded the known product **21** as a colorless oil in 80% yield (0.69 mmol; 155 mg) [2].

^{1}H NMR ($CDCl_3$, 300 MHz)**:** 0.82–0.93 (m, 3H, H_{10}); 0.99 (d, *J* = 6.6 Hz, 3H, H_{12}); 1.20–1.40 (m, 10H, $H_{5\text{-}9}$); 1.30 (t, *J* = 7.1 Hz, 3H, H_{14}); 1.83 (d, *J* = 1.3 Hz, 3H, H_{11}); 2.41–2.56 (m, 1H, H_4); 4.18 (q, *J* = 7.2 Hz, 2H, H_{13}); 6,53 (dq, ^3J = 10.1 Hz, ^4J = 1.4 Hz, 1H, H_3). **^{13}C NMR** ($CDCl_3$, 75 MHz)**:** 12.6–14.2–14.4–20.1 (q, 4C, $C_{10\text{-}12,14}$); 22.8–27.6–29.5–31.9–37.0 (t, 5C, $C_{5\text{-}9}$); 33.4 (d, C_4); 60.5 (t, C_{13}); 126.4 (s, C_2); 148.3 (d, C_3); 168.6 (s, C_1). **$[\alpha]^{22}_D$** = −18.7 (c 0.5; $CHCl_3$). Literature: **$[\alpha]^{22}_D$** = −25.9 (c 0.75; CH_2Cl_2) [4].

(*4R*)-2,4-dimethyl-2-decen-1-ol: 22 [2].

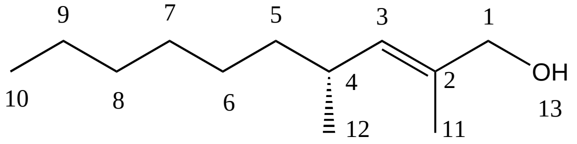

To a solution of **21** (0.35 mmol; 80 mg) in dry THF (3 mL) cooled down to 0 °C, under an argon atmosphere, was added dropwise the DIBAL-H 1M in heptane (0.74 mmol; 0.74 mL; 2.1 equiv). The solution was stirred to rt for 2 h before being quenched by adding 1N HCl until pH = 1. The white precipitate formed was removed by filtration and the filtrate was extracted with DCM (3 × 10 mL). The combined organic extracts were dried over $MgSO_4$, filtered and concentrated in vacuo to afford the known product **22** as a colorless oil in quantitative yield (0.35 mmol; 64 mg).

^{1}H NMR ($CDCl_3$, 300 MHz)**:** 0.84–0.95 (m, 3H, H_{10}); 0.93 (d, *J* = 6.8 Hz, 3H, H_{12}); 1.16–1.36 (m, 10H, $H_{5\text{-}9}$); 1.66 (d, *J* = 1.3 Hz, 3H, H_{11}); 2.28–2.43 (m, 1H, H_4); 3.99 (d, *J* = 0.9 Hz, 2H, H_1); 5.17 (dq, ^3J=9.5 Hz, ^4J = 1.3 Hz, 1H, H_3).

(*4R*)-2,4-dimethyl-2-decenal: 23

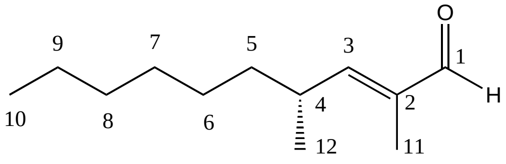

To a solution of **22** (0.35 mmol; 65 mg) in dry DCM (4 mL) at 0 °C was added the Dess-Martin periodinane 15% weight in DCM (0.42 mmol; 0.87 mL; 1.2 equiv). The reaction was allowed to stir to rt for 2 h and was then quenched by adding a 1:1 mixture of saturated aqueous NH_4Cl:$Na_2S_2O_3$. The aqueous layer was extracted with DCM (3 × 10 mL) and the combined organic extracts were dried over $MgSO_4$, filtered and concentrated in vacuo. Flash chromatography (95:5 petroleum ether:EtOAc) afforded product **23** as a colorless oil in 75% yield (0.26 mmol; 47.6 mg).

^{1}H NMR ($CDCl_3$, 300 MHz)**:** 0.84–0.92 (m, 3H, H_{10}); 1.06 (d, *J* = 6.8 Hz, 3H, H_{12}); 1.18–1.35 (m, 10H, $H_{5\text{-}9}$); 1.75 (d, *J* = 1.3 Hz, 3H, H_{12}); 2.60–2.76 (m, 1H, H_4); 6.25 (dq, ^3J = 9.8 Hz, ^4J = 1.1 Hz, 1H, H_3); 9.39 (s, 1H, H_1).

(*6R*)-Ethyl 4,6-dimethyl-2,4-dodecadienoate: 24[5]

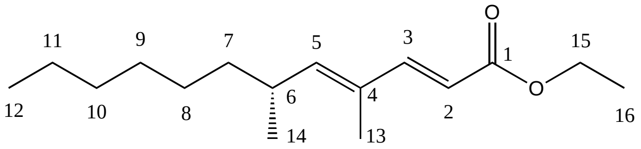

Triethyl phosphonoacetate **13c** (0.34 mmol; 90 µL; 1.2 equiv) was added dropwise to a suspension of NaH 60% weight in mineral oil (0.34 mmol; 13.5 mg; 1.2 equiv) in dry THF (2 mL) at 0 °C under a nitrogen atmosphere. After 1 h stirring, aldehyde **23** (0.28 mmol; 51 mg) was added dropwise. The reaction was stirred to rt overnight before being quenched with a saturated aqueous NH_4Cl solution. The solvent was removed by half in vacuo and the resulting mixture was extracted with DCM (3 × 5 mL). The combined organic extracts were dried over $MgSO_4$, filtered and concentrated in vacuo. Flash chromatography (98:2 petroleum ether:EtOAc) afforded the desired product **24** as a colorless oil in 77% yield (0.21 mmol; 55 mg).

^{1}H NMR ($CDCl_3$, 300 MHz)**:** 0.80–0.96 (m, 3H, H_{12}); 0.97 (d, *J* = 6.6 Hz, 3H, H_{14}); 1.15–1.50 (m, 13H, $H_{7\text{-}11,16}$); 1.77 (d, *J* = 1.14 Hz, 3H, H_{13}); 2.38–2.56 (m, 1H, H_6); 4.14 (q, *J* = 7.1 Hz, 2H, H_{15}); 5.67 (d, *J* = 9.8 Hz, 1H, H_5); 5.78 (d, *J* = 15.8 Hz, 1H, H_2); 7.31 (d, *J* = 15.8 Hz, 1H, H_3). Data in accordance with [5]

(*6R*)-4,6-Dimethyl-2,4-dodecadienoic acid: 25 [5]

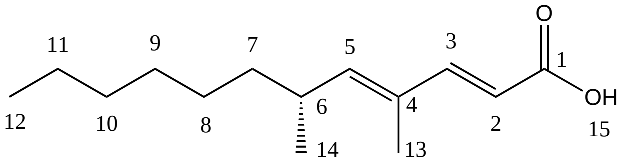

To a solution of **24** (0.21 mmol; 55 mg) in a 1:1:1 mixture of THF:MeOH:H_2O (2 mL) was added LiOH.H_2O (2.1 mmol; 88 mg; 10 equiv). The reaction was stirred at rt overnight before being concentrated by half in vacuo, diluted with ether and washed with a saturated aqueous Na_2CO_3 solution (4 × 5 mL). The combined aqueous layers were acidified with 1N HCl until pH = 1 and was then extracted with EtOAc (5 × 5 mL). The organic extracts were dried over $MgSO_4$, filtered and concentrated in vacuo, affording the desired product **25** as a pale yellow oil in 70% yield (0.15 mmol; 34 mg).

^{1}H NMR ($CDCl_3$, 300 MHz)**:** 0.80–0.94 (m, 3H, H_{12}); 0.99 (d, *J* = 6.6 Hz, 3H, H_{14}); 1.15–1.43 (m, 10H, $H_{7\text{-}11}$); 1.79 (d, *J*=1.2 Hz, 3H, H_{13}); 2.47–2.59 (m, 1H, H_6); 5.76 (d, *J* = 9.9 Hz, 1H, H_5); 5.82 (d, *J* = 15.6 Hz, 1H, H_2); 7.42 (d, *J* = 15.6 Hz, 1H, H_3). Data in accordance with reference [5].

O-t-Butyldimethylsilyl L-tyrosine methylester: 26 [6]

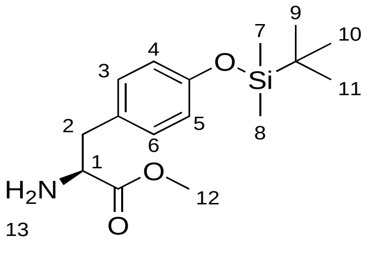

To a solution of L-tyrosine methylester (0.51 mmol; 100 mg) in dry DCM (5 mL) were added imidazole (0.61 mmol; 93 mg; 1.2 equiv) and TBSCl (0.61 mmol; 42 mg; 1.2 equiv). The reaction was stirred at rt overnight before being quenched with a saturated aqueous NH_4Cl solution. Phases were separated and the aqueous layer was extracted with DCM (3 × 5 mL). The organic extracts were dried over $MgSO_4$, filtered and concentrated in vacuo. Flash chromatography (EtOAc) afforded the desired product **26** as a colorless oil in 87% yield (0.21 mmol; 137 mg) whose data were in accordance with the literature [6].

Methyl 2-[(*2E,4E*)-4,6-dimethyldodeca-2,4-dienamido]-3-(4-*t*-butyldimethylsilyloxyphenyl)-propanoate : 27

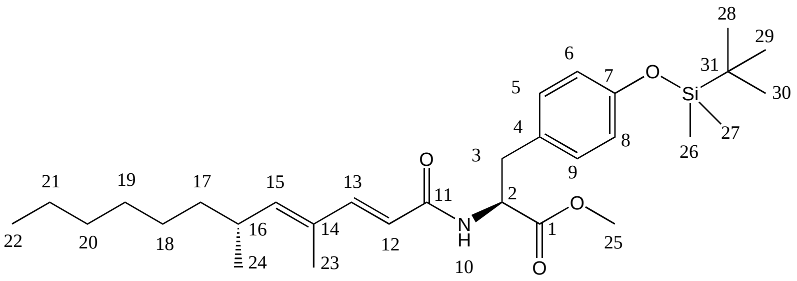

To a solution of acid **25** (0.15 mmol; 34 mg), TBS-protected L-tyrosine methylester **26** (0.17 mmol; 53 mg; 1.1 equiv) and HOBt (0.17 mmol; 23 mg; 1.1 equiv) in DCM (2 mL) was added DCC (0.17 mmol; 35 mg; 1.1 equiv). The reaction was stirred at rt for 5.5 h. The solvent was removed in vacuo and flash chromatography of the residue (85:15 petroleum ether:EtOAc) afforded the desired product **27** as a colorless oil in 57% yield (85 μmol; 44 mg).

^{1}H NMR (CDCl$_3$, 500 MHz)**:** 0.17 (s, 6H, $H_{26,27}$); 0.87 (t, J = 6.7 Hz, 3H, H_{22}); 0.96 (d, J = 6.6 Hz, 3H, H_{24}); 0.97 (s, 9H, H_{28-30}); 1.12–1.42 (m, 10H, H_{17-21}); 1.74 (d, J = 0.9 Hz, 3H, H_{23}); 2.43–2.55 (m, 1H, H_{16}); 3.09 (dd, J_1 = 5.6 Hz, J_2 = 2.0 Hz, 2H, H_3); 3.70 (s, 3H, H_{25}); 4.94 (dt, J_1 = 7.7 Hz, J_2 = 5.7 Hz, 1H, H_2); 5.62 (d, J = 9.8 Hz, 1H, H_{15}); 5.73 (d, J = 15.3 Hz, 1H, H_{12}); 5.97 (d, J = 7.8 Hz, 1H, H_{10}); 6.75 (d, J = 8.4 Hz, 2H, $H_{6,8}$); 6.95 (d, J = 8.4 Hz, 2H, $H_{4,9}$); 7.21 (d, J = 15.3 Hz, 1H, H_{13}). **^{13}C NMR** (CDCl$_3$, 75 MHz)**:** 4.3; 12.6; 14.2. 18.3; 20.7; 22.8; 25.8; 27.6; 29.5; 32.0; 37.3; 37.4; 52.4; 53.4; 117.4; 120.2; 128.7; 130.4; 131.0; 147.1; 148.0; 154.8; 166.2; 172;4.

Gymnastatin H: 10c [7]

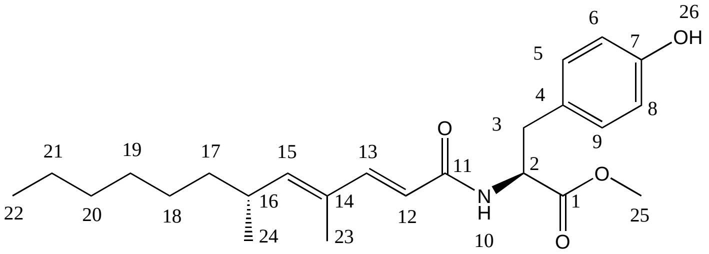

To a solution of **27** (77.5 μmol; 40 mg) in dry THF (1 mL) was added TBAF 1M in THF (85.3 μmol; 85 μL; 1.1 equiv). The solution was stirred at rt for 3 h before being quenched with a saturated aqueous NH_4Cl solution. The resulting mixture was extracted with DCM (3 × 5 mL). The organic extracts were dried over $MgSO_4$, filtered and concentrated in vacuo. Flash chromatography (50:50 petroleum ether:EtOAc) afforded gymnastatin H **10c** as a colorless oil in 96% yield (75 μmol; 30 mg).

^{1}H NMR (CDCl$_3$, 500 MHz)**:** 0.87 (t, J = 6.7 Hz, 3H, H_{22}); 0.95 (d, J = 6.6 Hz, 3H, H_{24}); 1.18–1.33 (m, 10H, H_{17-21}); 1.73 (d, J = 0.7Hz, 3H, H_{23}); 2.41–2.55 (m, 1H, H_{16}); 3.10 (dd, J_1 = 14.1 Hz, J_2 = 5.8 Hz, 1H, H_{3a}); 3.03 (dd, J_1 = 14.1 Hz, J_2 = 5.8 Hz, 1H, H_{3b}); 3.72 (s, 3H, H_{25}); 4.96 (dt, J_1 = 7.9 Hz, J_2 = 5.7 Hz, 1H, H_2); 5.62 (d, J = 9.8 Hz, 1H, H_{15}); 5.74 (d, J = 15.3 Hz, 1H, H_{12}); 6.11 (d, J = 8.0 Hz, 1H, H_{10}); 6.74 (d, J = 8.5 Hz, 2H, $H_{6,8}$); 6.94 (d, J = 8.5 Hz, 2H, $H_{5,9}$); 7.23 (d, J = 15.3 Hz, 1H, H_{13}). **^{13}C NMR** (CDCl$_3$, 75 MHz)**:** 12.6; 14.2; 20.6; 22.8; 27.6; 29.5; 31.9; 33.3; 37.4; 52.5; 53.6; 115.7; 117.0; 127.2; 130.4; 131.0; 147.6; 148.5; 155.6; 166.7; 172.6. **$[\alpha]^{22}_D$** = +104 (c 0.3; CHCl$_3$) – Lit.: **$[\alpha]^{22}_D$** = +42.3 (c 0.14; CHCl$_3$). **HRMS (ESI):** Calculated : M + Na$^+$: 424.2458; Found: M + Na$^+$: 424.2451. Spectroscopic data in accordance with [7].

References

1. Nagumo, S.; Miura, T.; Mizukami, M.; Miyoshi, I.; Imai, M.; Kawahara, N.; Akita, H. *Tetrahedron* **2009,** *65,* 9884–9896. doi:10.1016/j.tet.2009.09.037
2. Wipf, P.; Kim, Y.; Fritch, P. C. *J. Org. Chem.* **1993,** *58,* 7195–7203. doi:10.1021/jo00077a050

3. Goldstein, S. W.; Overman, L. E.; Rabinowitz, M. H. *J. Org. Chem.* **1992,** *57,* 1179–1190. doi:10.1021/jo00030a026
4. McKillop, A.; McLaren, L.; Watson, R. J.; Taylor, R. J. K.; Lewis, N. *Tetrahedron Lett.* **1993,** *34,* 5519–5522. doi:10.1016/S0040-4039(00)73870-6
5. Alcaraz, L.; Macdonald, G.; Ragot, J.; Lewis, N. J.; Taylor, R. J. K. *Tetrahedron* **1999,** *55,* 3707–3716. doi:10.1016/S0040-4020(98)00879-5
6. Jacobsen, E. J.; Mitchell, M. A.; Hendges, S. K.; Belonga, K. L.; Skaletzky, L. L.; Stelzer, L. S.; Lindberg, T. J.; Fritzen, E. L.; Schostarez, H. J.; O'Sullivan, T. J.; Maggiora, L. L.; Stuchly, C. W.; Laborde, A. L.; Kubicek, M. F.; Poorman, R. A.; Beck, J. M.; Miller, H. R.; Petzold, G. L.; Scott, P. S.; Truesdell, S. E.; Wallace, T. L.; Wilks, J. W.; Fisher, C.; Goodman, L. V.; Kaytes, P. S.; Ledbetter, S. R.; Powers, E. A.; Vogeli, G.; Mott, J. E.; Trepod, C. M.; Staples, D. J.; Baldwin, E. T.; Finzel, B. C. *J. Med. Chem.* **1999,** *42,* 1525–1536. doi:10.1021/jm9803222
7. Amagata, T.; Minoura, K.; Numata, A. *J. Nat. Prod.* **2006,** *69,* 1384–1388. doi:10.1021/np0600189
